# Strontium enhances osseointegration of calcium phosphate cement: a histomorphometric pilot study in ovariectomized rats

**DOI:** 10.1186/1749-799X-8-16

**Published:** 2013-06-07

**Authors:** Martin Baier, Patric Staudt, Roman Klein, Ulrike Sommer, Robert Wenz, Ingo Grafe, Peter Jürgen Meeder, Peter P Nawroth, Christian Kasperk

**Affiliations:** 1 Division of Traumatology, University of Heidelberg, Schlierbacher Landstraße 200a, Heidelberg 69118, Germany; 2 Division of Osteology, Department of Medicine I and Clinical Chemistry, University of Heidelberg, Im Neuenheimer Feld 410, 69120, Heidelberg, Germany; 3 Medtronic GmbH, Earl-Bakken-Platz 1, Meerbusch 40670, Germany

**Keywords:** Strontium, Calcium phosphate cement, Osteogenesis, Bone healing, Osteoporosis

## Abstract

**Background:**

Calcium phosphate cements are used frequently in orthopedic and dental surgeries. Strontium-containing drugs serve as systemic osteoblast-activating medication in various clinical settings promoting mechanical stability of the osteoporotic bone.

**Methods:**

Strontium-containing calcium phosphate cement (SPC) and calcium phosphate cement (CPC) were compared regarding their local and systemic effects on bone tissue in a standard animal model for osteoporotic bone. A bone defect was created in the distal femoral metaphysis of 60 ovariectomized Sprague-Dawley rats. CPC and SPC were used to fill the defects in 30 rats in each group. Local effects were assessed by histomorphometry at the implant site. Systemic effects were assessed by bone mineral density (BMD) measurements at the contralateral femur and the spine.

**Results:**

Faster osseointegration and more new bone formation were found for SPC as compared to CPC implant sites. SPC implants exhibited more cracks than CPC implants, allowing more bone formation within the implant. Contralateral femur BMD and spine BMD did not differ significantly between the groups.

**Conclusions:**

The addition of strontium to calcium phosphate stimulates bone formation in and around the implant. Systemic release of strontium from the SPC implants did not lead to sufficiently high serum strontium levels to induce significant systemic effects on bone mass in this rat model.

## Background

Implant materials on the basis of calcium phosphate are frequently used with the goal of complete osseointegration and eventual substitution by the bone tissue [1-3]. Factors modulating bone formation are added to some implant materials to enhance osseointegration. The release of these factors occurs either spontaneously or as a consequence of the bony replacement of the implant. There have been numerous reports on osseous integration of organic and inorganic implant materials releasing growth factors like bone morphogenetic protein 2 (BMP-2) [4-7], bone morphogenetic protein 7 (BMP-7) [8], fibroblast growth factor 2 (FGF-2) [9], platelet-derived growth factor [10], and insulin-like growth factors [11]. Some of these products are available for routine procedures. Although most of the published preclinical reports demonstrate a faster healing of osseous defects within the first 2 to 3 weeks, the clinical long-term relevance of these observations in animal experiments remains unclear.

Strontium administered systemically increases bone mass by stimulating osteoblastic activity [12-14] and simultaneously inhibiting bone resorption [15-17]. In preclinical experiments, oral administration of strontium ranelate prevented ovariectomy-induced bone loss in rats [15]. Subsequent clinical trials demonstrated a significant fracture prevention after strontium ranelate treatment of osteoporotic patients (TROPOS [18] and SOTI [19] studies).

We hypothesized that adding of strontium to calcium phosphate cement implants may improve osseous integration of the implant into the osseous implant bed and may also increase bone formation locally at the implanted skeletal site and systemically at the contralateral skeletal site and at the spine.

Osseointegration-stimulating properties of a bone substitute are of particular interest in an osteoporotic skeleton, which does not provide an optimal bone structure for surgical implantation procedures. To address this problem, we chose the ovariectomized rat model [20,21] which is the standard animal model for the histomorphometric evaluation of bone healing in the osteoporotic bone.

The goals of our study were to assess osseointegration, compare calcium phosphate cement implants containing strontium (SPC) to calcium phosphate cement implants (CPC), and to determine whether a locally applied strontium-containing implant has systemic effects on bone mass.

## Materials and methods

### Animal experiments

The animal experiment was conducted in accordance with the guidelines set forth by the local animal protection committee of the regional government (Regierungspräsidium Karlsruhe, file number AZ 35-9185.82/A-49/04).

A standard animal model [20] for osteoporosis was used: 60 female Sprague-Dawley rats were ovariectomized at the age of 10 weeks under anesthesia with intraperitoneal injection of 100 mg/kg ketamine and 3 mg/kg xylazine and subcutaneous injection of carprofen (all three chemicals supplied by Pfizer, 76139 Karlsruhe, Germany).

Two months after ovariectomy, osteopenia was assessed by dual energy X-ray absorptiometry (Piximus device by GE, Fairfield, CT, USA). Successful ovariectomy-induced loss of bone mass was verified by measuring bone mineral density at the left femur (distal metaphysis) which exhibited a decrease of bone mineral density by 12% after 3 months as evaluated by the comparison to intact controls (data not shown).

*Surgical procedure*. In anesthesia, the right distal femur was exposed through a lateral approach. With a 2-mm drill, a bone defect was created proximal to the distal femoral growth line in the lateral femoral cortex and the metaphyseal cancellous bone. The left femur was not operated on and served as control.

**Figure 1:**
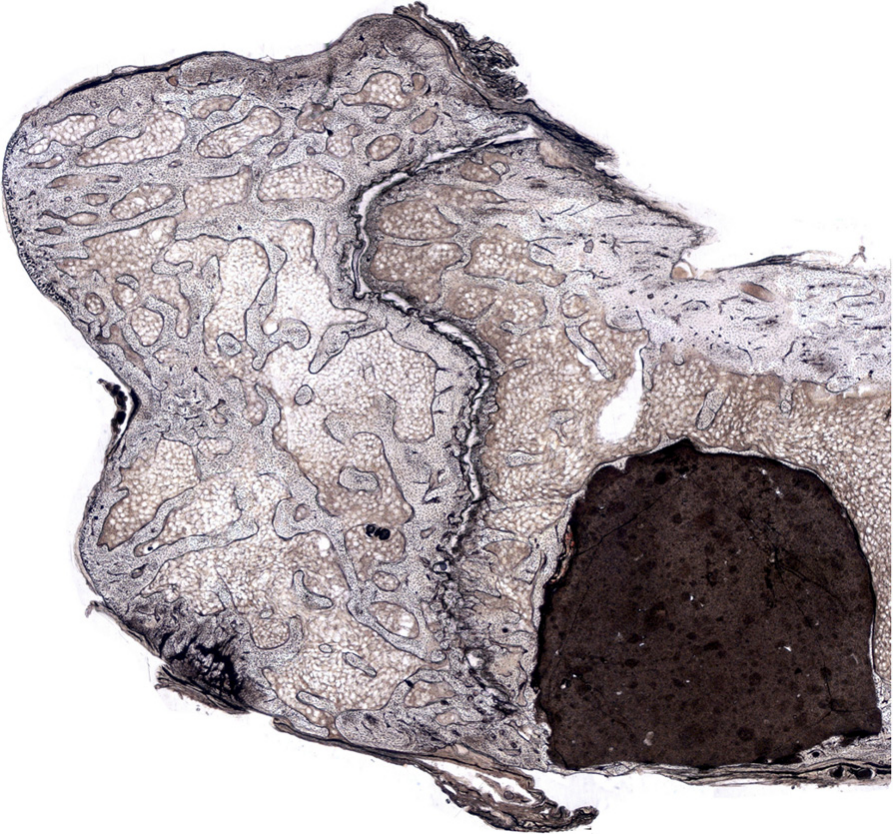
**Calcium phosphate implant in rat femur after implantation.** Section of a representative specimen of the distal right femur with cement implant (CPC, 3 months post implantation).

In 30 animals, the defect was filled with 0.25 ml of CPC and in another 30 animals with the same volume of SPC (Figure 1).

### Cement

### Calcium phosphate cement

The solid phase of CPC consisted of 61% tricalcium phosphate (TCP), 26% calcium hydrogen phosphate (CaHPO_4_, anhydrous), 10% calcium carbonate (CaCO_3_), and 3% precipitated hydroxyapatite (pHA). The liquid phase of CPC was an aqueous solution of 2% disodium hydrogen phosphate (Na_2_HPO_4_). The compression strength of CPC was 60 MPa (24 h after preparation).

### Strontium-containing calcium phosphate cement

The solid phase of SPC consisted of 65.2% tricalcium phosphate (TCP), 21.8% strontium hydrogen phosphate (SrHPO_4_, anhydrous), 10.8% strontium carbonate (SPCO_3_), and 2.2% precipitated hydroxyapatite (pHA). The liquid phase of SPC was an aqueous solution of 3 M dipotassium hydrogen phosphate (K_2_HPO_4_) and 1.5 M potassium dihydrogen phosphate (KH_2_PO_4_), with a volume ratio of 1:1. The compression strength of SPC was 34 MPa (24 h after preparation).

### Histomorphometric evaluation

After embedding the specimen in polymethylmethacrylate (PMMA), the slices for histological evaluation were gained by slicing the PMMA blocks containing undecalcified bone specimen with a diamond band saw (Exakt GmbH, Norderstedt, Germany) and subsequent grinding of the slices to a thickness of 100 μm. The thin ground sections were mounted on plastic slides and photographed digitally with 25-fold magnification. Measurements were performed with AxioVision software (Carl Zeiss Vision GmbH, Oberkochen, Germany). The following histomorphometric parameters were determined:

### Circumferential contact index

**Figure 2:**
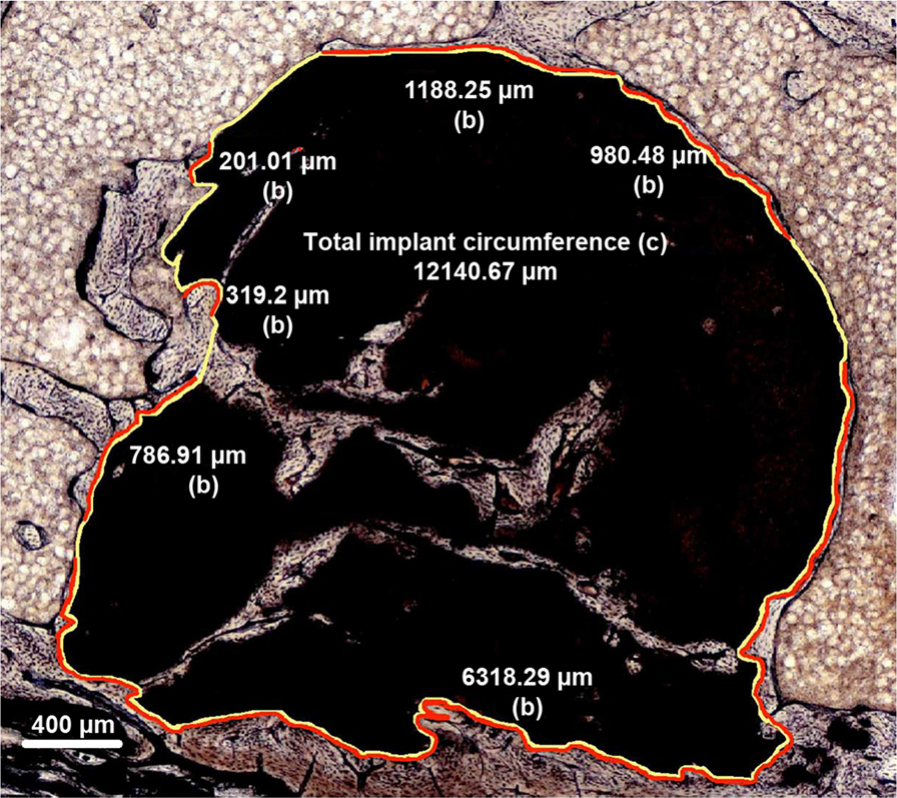
**Circumferential contact index.** Example of SPC, 6 months after implantation): On the outer surface of the implant, the length of all circumferential segments with direct contact between cement and bone (b = sum of all red lines) as well as the total implant circumference (c) were measured. Circumferential contact index was defined as (*b* / *c* × 100 (%)).

Implant circumference (c in Figure 2) and the length of all circumferential segments with direct contact between implant and bone (b in Figure 2) were measured. Circumferential contact index was defined as (*b* / *c* × 100 (%)). This parameter was determined at 1, 3, and 6 months after implantation. Contact segments of newly formed bone within the implant were ignored in order to keep this parameter independent from the other parameters mentioned below (Figure 2).

### Ingrowth index

**Figure 3:**
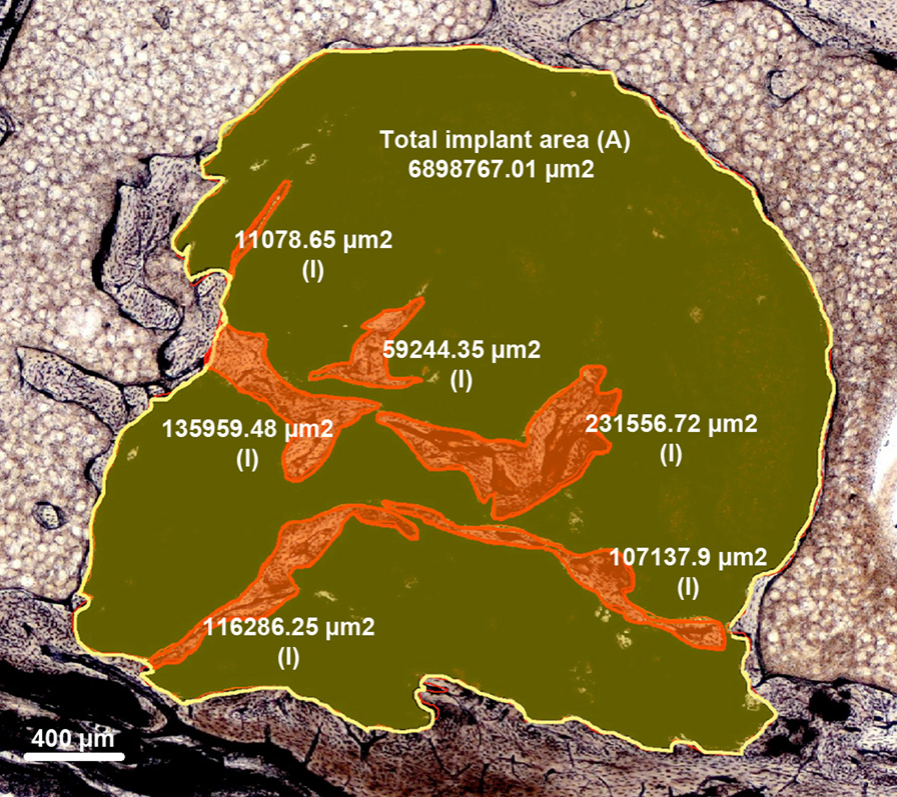
**Ingrowth index.** The relative area of newly formed bone within the implant was measured (SPC, 6 months after implantation). Ingrowth index was defined as (*I* / *A* × 100 (%)).

Cement implant area (A in Figure 3) and the area of newly formed bone within the implant (I in Figure 3) were measured. Ingrowth index was defined as (*I* / *A* × 100 (%)). This parameter was determined at 1, 3, and 6 months after implantation (Figure 3).

### Implant discontinuities

**Figure 4:**
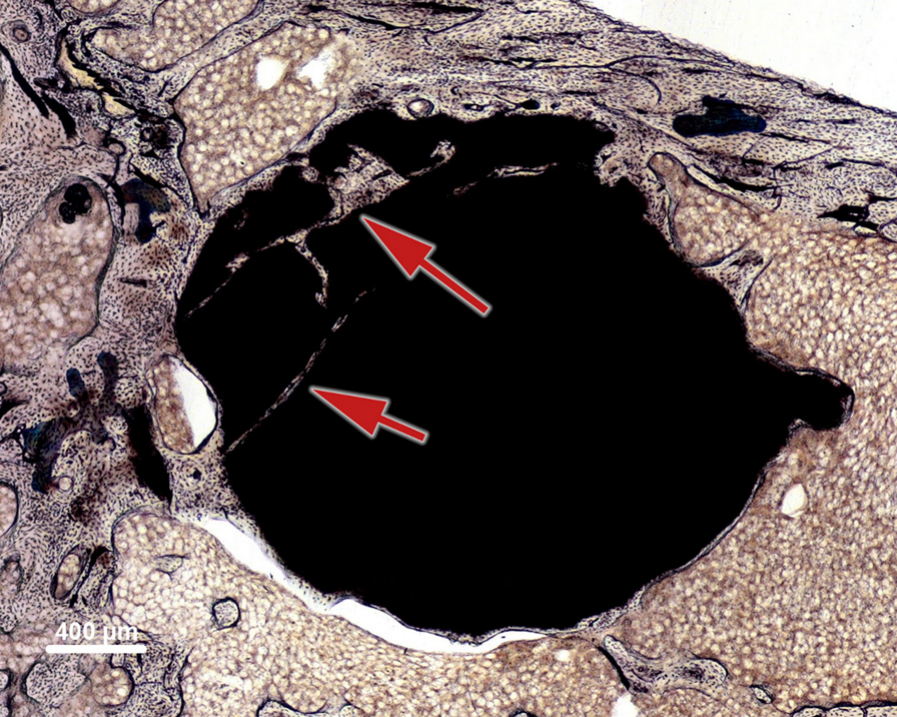
**Number of discontinuities.** The total number of discontinuities was counted: This specimen has two implant discontinuities (*red arrows*) containing newly formed bone (SPC, 6 months after implantation).

The total number of discontinuities within the implant and the number of discontinuities containing newly formed bone were counted at 1, 3, and 6 months after implantation. Only discontinuities with a length greater than half of the implant diameter were considered (Figure 4).

### Evaluation of systemic effects

### Bone mineral density measurement

In order to evaluate whether locally applied SPC or CPC in the right femur differ in their potential systemic effects on bone mineral density (BMD), BMD was measured with dual X-ray absorptiometry (Piximus device by GE) in the distal metaphysis of the left femur and in the cancellous bone of the second coccygeal vertebra.

### Serum strontium levels

Blood samples of all animals with SPC implants were taken directly after killing. Serum levels of strontium were measured by mass spectrometry.

### Statistical analysis

The Mann-Whitney *U* test (two-sided, with significance level of *p* < 0.05) was applied to compare CPC and SPC groups at equal time points (1, 3, and 6 months after implantation). An overview of sample sizes is given in Table 1.

**Table 1:** Sample sizes for statistical analyses

	**Histological measurement**		**BMD measurement**	
	**CPC (*n*)**	**SPC (*n*)**	**CPC (*n*)**	**SPC (*n*)**
1 month	8	9	10	9
3 months	8	10	9	10
6 months	9	7	9	9

Of the 60 animals distributed into six groups of ten animals each (*CPC* and *SPC* at 1, 3, and 6 months), some got lost for *BMD* and/or histological measurement. In the 1-month group, two *CPC* implants disintegrated during preparation, and one animal with *SPC* had died earlier for unknown reasons. In the 3-month group, one *CPC* implant disintegrated during preparation, and one animal with *CPC* had died earlier for unknown reasons. In the 6-month group, two *SPC* implants disintegrated during preparation, and one animal with *CPC* and one with *SPC* had died earlier for unknown reasons.

## Results

### Histomorphometric parameters

### Circumferential contact index

**Figure 5:**
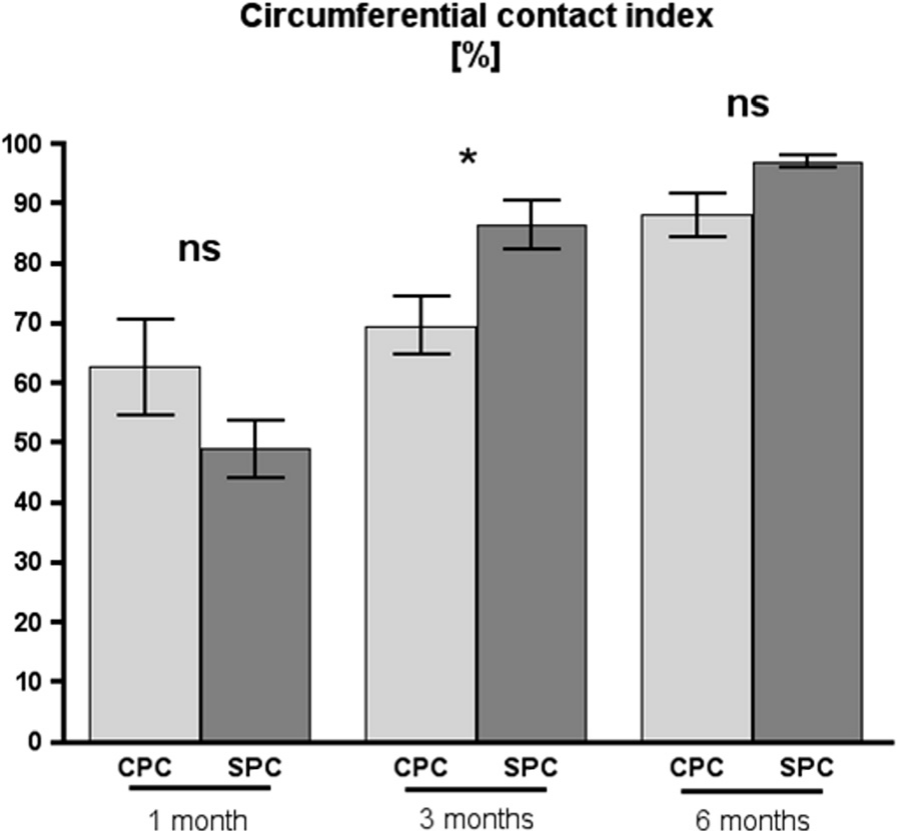
**Circumferential contact index.** Circumferential contact index for calcium phosphate cement (CPC) and strontium-containing calcium phosphate cement (SPC) at 1, 3, and 6 months after implantation. Three months after implantation, the circumferential contact index is significantly higher for SPC as compared to CPC (*p* = 0.009, as indicated by *double asterisk*) (mean values ± standard error, using two-sided Mann-Whitney *U* test for comparison between CPC and SPC at equal points in time with significance level at *p* < 0.05).

One month after implantation, the circumferential contact index did not differ significantly between the CPC and SPC groups. At 3 and 6 months, circumferential contact index was higher in the SPC group as compared to the CPC group; however, after 6 months this difference was not significant anymore. In both groups, SPC and CPC, the circumferential contact index increased over time (Figure 5).

### Ingrowth index

**Figure 6:**
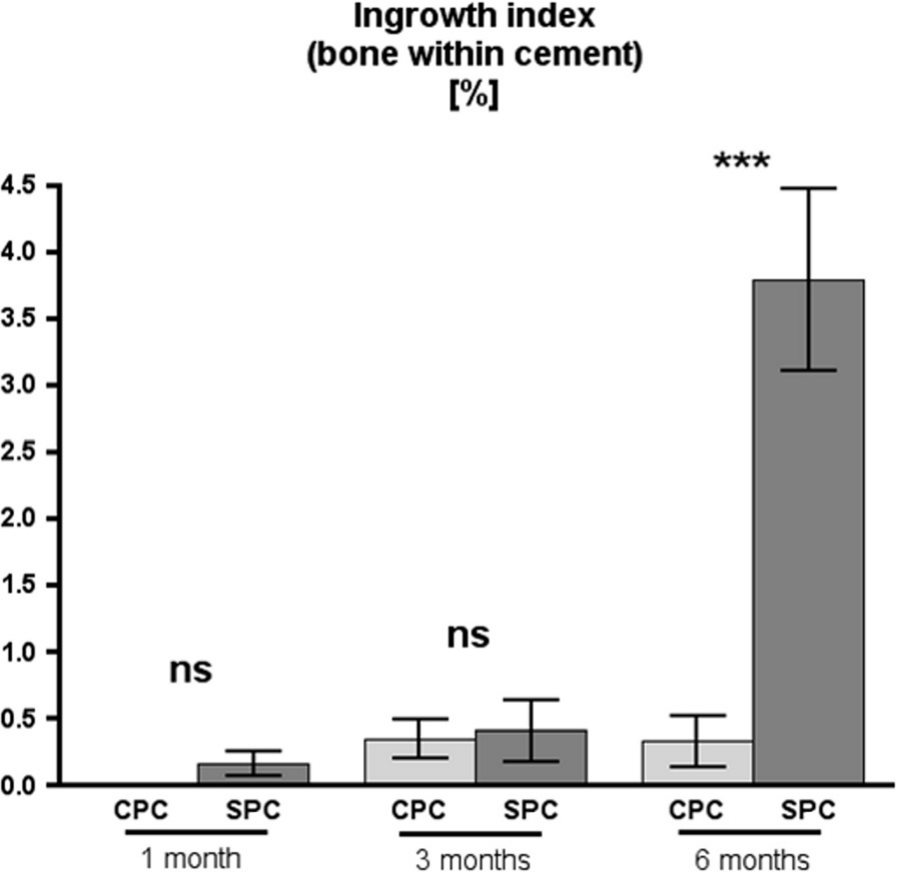
**Ingrowth index for CPC and SPC at 1, 3, and 6 months after implantation.** At 6 months, ingrowth index is significantly higher for SPC as compared to CPC (*p* = 0.0002, as indicated by *triple asterisks*) (mean values ± standard error, using two-sided Mann-Whitney *U* test for comparison between CPC and SPC at equal points in time with significance level at *p* < 0.05).

One month and three months after implantation, the ingrowth index did not differ significantly between the CPC and SPC groups. At 6 months, both groups differed in a highly significant way: While ingrowth index in the CPC group was only 0.33%, it was 3.79% in the SPC group (*p* = 0.0002) (Figure 6), indicating that after 6 months, a higher percentage of more than ten times in intra-implant bone formation occurred within the SPC implants.

### Implant discontinuities

**Figure 7:**
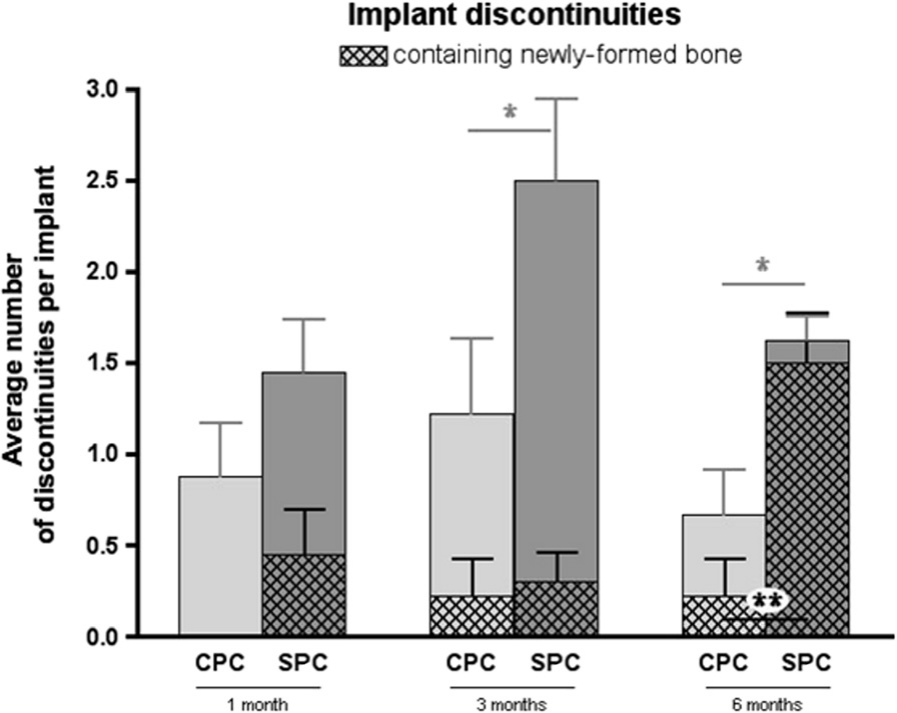
**Implant discontinuities.** Number of implant discontinuities and number of implant discontinuities containing newly formed bone at 1, 3, and 6 months post-implantation. Three months and six months post-implantation, SPC has significantly more discontinuities than CPC. Six months post-implantation, the number of discontinuities containing the newly formed bone is significantly higher in SPC as compared to CPC (mean values ± standard error, using two-sided Mann-Whitney *U* test for comparison between CPC and SPC at equal points in time with significance level at *p* < 0.05).

There were significantly more discontinuities in SPC implants as compared to CPC implants. This was statistically significant at 3 and 6 months after implantation (Figure 7).

In both types of cement, the presence of newly-formed bone within these discontinuities increased over time. This effect was much more pronounced in SPC than in CPC implants. Six months after implantation, SPC implants had a significantly higher number of bone-containing discontinuities (on average of 1.5 in SPC and 0.2 in CPC; *p* = 0.0079, indicated by double asterisks) (Figure 7).

### Systemic parameters

### Bone mineral density

BMD results are shown in Table 2. No significant difference was found between CPC and SPC groups. However, in the SPC group, there was a consistent increase of bone mineral density in the distal metaphyses of the left (contralateral to the femoral implant site) femora during the observation period.

**Table 2:** Bone mineral density during 6 months of observation

	**Coccygeal vertebrae**		**Left femora**	
	**CPC**	**SPC**	**CPC**	**SPC**
1 month	0.261 ± 0.020	0.274 ± 0.025	0.257 ± 0.015	0.254 ± 0.038
3 months	0.261 ± 0.021	0.256 ± 0.031	0.262 ± 0.016	0.267 ± 0.018
6 months	0.264 ± 0.012	0.266 ± 0.026	0.256 ± 0.019	0.275 ± 0.026

Bone mineral density (g/cm^2^) of the second coccygeal vertebrae and at the distal metaphyses of the left femora 1, 3, and 6 months after implantation (arithmetic mean ± standard deviation).

### Serum strontium levels

Serum strontium concentrations (mean ± standard deviation) in the SPC group at 1, 3, and 6 months were 10.87 ± 4.16 μg/l, 3.73 ± 0.88 μg/l, and 1.80 ± 0.39 μg/l, respectively.

## Discussion

### Local effects of strontium-containing cement

This work demonstrates that the addition of strontium to calcium phosphate cement enhances local bone formation in ovariectomized rats, both on the implant surface and within the implant. *In vitro* experiments provide possible explanations for the observed bone formation stimulating effect of locally applied strontium. Divalent strontium ions enhance the replication of pre-osteoblastic cells and bone matrix synthesis by binding to the calcium receptor and stimulation of local growth factor and osteoprotegerin production [13,22,23].

The histomorphometric evaluation of the implants suggests that the degradation process of calcium phosphate implants is based on the formation of discontinuities within the implant, which are subsequently filled with newly formed bone. While the number of discontinuities in calcium phosphate implants remained constant over the observation period, the number of discontinuities gradually increased in strontium-containing implants. Bone formation within implant discontinuities was strongly influenced by the presence of strontium. More than nine out of ten discontinuities in strontium cement implants contained newly formed bone after 6 months, compared to only four out of ten discontinuities in calcium phosphate cement implants.

The physicochemical properties of the strontium-containing implant determine its osteotropic effects and its mechanical properties. The solubility of calcium phosphates in which part of the Ca^2+^ ions are replaced by Sr^2+^ ions increases with increasing content of strontium ions due to the larger atomic radius of Sr^2+^ which reduces crystallinity and alters the crystal lattice [24-27]. The higher solubility of the SPC compared to the CPC could also explain the higher degree of discontinuities within the SPC group, which may compromise the biomechanical stability of the implant.

Different types of strontium-containing cements have been tested *in vivo* previously: In rabbit non-osteoporotic cancellous bone, an injectable strontium-containing cement with an acrylate component was compared to PMMA cement [28]. The stimulation of bone formation at the bone-implant interface was found on strontium-containing cement surfaces only, whereas inflammatory responses, necrosis, and a fibrous layer were found at the PMMA-cement-bone interface [28]. Another calcium phosphate cement containing strontium and acrylate was developed by Lu, Cheung, and co-workers, which also exhibited direct contact between bone and strontium-containing cement [29,30]. Guo Dagang and co-workers [31,32] demonstrated biocompatibility and degradability of their strontium-containing hydroxyapatite in rabbit muscle and cancellous bone.

The addition of strontium to different types of cement also affects the compression strength of the material in different ways. Panzavolta et al. [23] describe a strontium-enriched gelatin-calcium phosphate cement: Its compressive strength decreases with increasing strontium content (0.1% to 5%). However, Wang et al. [33], who added strontium carbonate as a radio-opacifier to calcium phosphate cement, observed an increase in compressive strength from 0 wt.% to 8 wt.% strontium carbonate but with a subsequent decrease in compressive strength when increasing the strontium carbonate content to 20 wt.%. The absolute figure for compression strength for cement with 20 wt.% strontium [31-33] was similar to the one in our study (36 MPa as compared to SPC used in our study with 34 MPa). It is unclear how mechanical cement properties affect the degradation processes in bone tissue. Cements implanted into a cavity surrounded by femoral bone - as performed in our study - are somewhat shielded from mechanical loading. Despite this shielding, compressive forces and shear stress within the implant are possibly due to the elastic deformation of the surrounding bone when the animal puts weight on the femur when moving. It is therefore likely that formation of discontinuities and eventually cement degradation is influenced by the mechanical properties of the cement, which may also have clinical implications when calcium phosphate cements are implanted in bone sites exposed to shear stress rather than compressive force.

Unlike the local positive effects of strontium-containing cements on osseointegration, we did not find a significant systemic effect on bone density of strontium-containing cement implants. BMD in the examined vertebrae did not differ between animals with calcium phosphate and strontium-containing calcium phosphate implants in the right femur. In the contralateral femur, bone mineral density increased by approximately 8% in animals with strontium-containing cement from 1 to 6 months after implantation. No such increase was observed in the calcium phosphate group; however, the difference in BMD values between the SPC and CPC groups was not significant at any point in time. When considering these observations, we have to keep in mind a peculiarity of the used rat animal model. The ovariectomized rats' skeleton is still growing during the observation period, and the growth rate at the epiphyseal plates is usually high in ovariectomized rats [34]. If the serum levels of strontium are elevated shortly after implantation, part of the serum strontium is likely to be deposited in the growing bone. The more bone tissue is growing, the more strontium can be deposited into the newly formed bone matrix, and in fact higher concentrations of strontium were observed in newly formed bone than in old bone in other studies [35,36]. BMD measurements in strontium-containing bone are falsely high, due to the higher atomic number (*Z* = 38) of strontium as compared to calcium (*Z* = 20) [25,37,38]. The increase in femoral BMD over time while vertebral BMD remains unchanged may therefore be due to the differences in relative growth between the femur and spine during the observation period.

Another possible reason for increased contralateral femoral BMD while spinal BMD remains unaffected could be the different loading conditions of the respective skeletal sites. In the rat, mechanical load on the coccygeal vertebrae is lower compared to the load on a weight-bearing femur. A study which investigated an effect of different loading conditions on BMD under systemic application of strontium was performed by Hott et al. [39]: Bone resorption induced by immobilization in rats can be suppressed by systemic administration of strontium. Whether bone formation under application of strontium is possibly enhanced by mechanical loading or not has not been shown.

Oral administration of strontium ranelate increases bone mineral density in ovariectomized rats' femur, even after correction for higher X-ray absorption [15]. The lowest strontium ranelate dose in a rat experiment of 77 mg/kg/day orally led to a serum strontium concentration of 24.2 μg/l [15], which is more than twice the concentration we measured in the serum 1 month after implantation of the strontium-containing cement. The oral dose that raised BMD most in the study by Marie et al. [15] (308 mg/kg/day) led to a serum strontium concentration of 127.6 μg/l, which is an order of magnitude higher than our highest serum concentration. In another study, Marie et al. demonstrated a substantial improvement of mechanical properties of the fifth lumbar vertebra in ovariectomized rats treated with oral strontium ranelate (625 mg/kg/day) [40]. Ultimate strength was increased by 26% and energy to failure by 75%. To achieve this mechanical improvement, the maximum oral strontium dose of their previous study [15] was more than doubled. The serum strontium concentration necessary to produce systemic effects was approximately 100 times higher than the one measured in our study [41].

There are several shortcomings of our study. The mechanical properties of the utilized cements in this study were tested under *in vitro* conditions by the manufacturer. Thus, it is unclear whether the indicated compressive strengths of the two cement types in our study remain constant after *in vivo* implantation. Confounding factors are the presence of body fluids and possible mechanical stress by inserting instruments during the crystallization phase; the surgical application procedure may at least in part be responsible for implant discontinuities and thus play a role in the subsequent degradation processes, thereby influencing resorption and eventually replacement of the implant.

The cements used in our study are resorbable; however, due to the lack of a long-term follow-up, we do not know whether the used cements are indeed replaceable by bone tissue and to what extent.

The parameters ‘ingrowth index’ and ‘implant discontinuities containing bone’ are not independent of each other and thus describe the osseointegration characteristics of the used cements with different parameters. After 6 months, a large proportion of the bone tissue quantified by the ingrowth index was actually located within linear implant discontinuities. New bone formation on the circumference of the original implant (‘circumferential contact index’), however, has been quantified as a parameter which is independent from the ingrowth indices (ingrowth index and implant discontinuities containing bone).

When assessing the bone mineral density after application of the implant containing strontium, we did not correct the higher X-ray absorption of strontium. Our BMD measurement may therefore produce falsely high values. This must be taken into account in case our absolute values are to be compared with others. The interpretation of our results, however, remains unchanged even if we corrected for bone strontium content, as we did not detect any significant systemic effects of locally applied strontium-containing implants on BMD at other skeletal sites.

## Conclusions

The addition of strontium to calcium-phosphate cement leads to a faster osseointegration of the implant into the osteoporotic bone. Strontium-containing implants stimulate, at least initially, local bone formation and are partly replaced by bone within 6 months.

Serum concentrations of strontium caused by release from the strontium-containing implants in this model are too low to stimulate systemic bone formation and thus do not increase bone mineral density systemically.

The clinical relevance of the observation of an initially faster osseointegration of strontium-containing implants and its biomechanical implications still have to be demonstrated by a long-term, randomized, controlled clinical trial.

## Competing interests

RW is an employee of Medtronic GmbH. All other authors have no competing interest.

## Authors' contributions

MB carried out data evaluation and editing of the manuscript. PS, US, WR, and IG performed the animal experiments. RK is responsible for the editorial work. PJM and PPN are both involved in the supervision of this work. CK designed the project, performed the experiments and edited the manuscript. All authors read and approved the final manuscript.
